# *In-silico* prediction of drug targets, biological activities, signal pathways and regulating networks of dioscin based on bioinformatics

**DOI:** 10.1186/s12906-015-0579-6

**Published:** 2015-03-05

**Authors:** Lianhong Yin, Lingli Zheng, Lina Xu, Deshi Dong, Xu Han, Yan Qi, Yanyan Zhao, Youwei Xu, Jinyong Peng

**Affiliations:** College of Pharmacy, Dalian Medical University, Western 9 Lvshun South Road, Dalian, 116044 China; The First Affiliated Hospital of Dalian Medical University, Dalian, 116022 China; Research Institute of Integrated Traditional and Western Medicine of Dalian Medical University, Dalian, 116011 China

**Keywords:** Dioscin, Inverse docking technology, Bioinformatics, Pathway, Network

## Abstract

**Background:**

Inverse docking technology has been a trend of drug discovery, and bioinformatics approaches have been used to predict target proteins, biological activities, signal pathways and molecular regulating networks affected by drugs for further pharmacodynamic and mechanism studies.

**Methods:**

In the present paper, inverse docking technology was applied to screen potential targets from potential drug target database (PDTD). Then, the corresponding gene information of the obtained drug-targets was applied to predict the related biological activities, signal pathways and processes networks of the compound by using MetaCore platform. After that, some most relevant regulating networks were considered, which included the nodes and relevant pathways of dioscin.

**Results:**

71 potential targets of dioscin from humans, 7 from rats and 8 from mice were screened, and the prediction results showed that the most likely targets of dioscin were cyclin A2, calmodulin, hemoglobin subunit beta, DNA topoisomerase I, DNA polymerase lambda, nitric oxide synthase and UDP-N-acetylhexosamine pyrophosphorylase, etc. Many diseases including experimental autoimmune encephalomyelitis of human, temporal lobe epilepsy of rat and ankylosing spondylitis of mouse, may be inhibited by dioscin through regulating immune response alternative complement pathway, G-protein signaling RhoB regulation pathway and immune response antiviral actions of interferons, etc. The most relevant networks (5 from human, 3 from rat and 5 from mouse) indicated that dioscin may be a TOP1 inhibitor, which can treat cancer though the cell cycle– transition and termination of DNA replication pathway. Dioscin can down regulate EGFR and EGF to inhibit cancer, and also has anti-inflammation activity by regulating JNK signaling pathway.

**Conclusions:**

The predictions of the possible targets, biological activities, signal pathways and relevant regulating networks of dioscin provide valuable information to guide further investigation of dioscin on pharmacodynamics and molecular mechanisms, which also suggests a practical and effective method for studies on the mechanism of other chemicals.

## Background

Bioinformatics uses statistics and computer science to process heterogeneous biological data, which provides opportunities for understanding disease genetics, biological processes and identifying therapeutic targets [1]. During the last decades, drug discoveries have tuned into the combination of experimental approaches and modern science of computational. Various tools and techniques have been used for target identification, enrichment analysis and network algorithm. To date, several *in silico* bioinformatic methods have been developed and applied [2,3]. Drug targets have been predicted by using chemical two-dimensional structural similarity approach and Bipartite graph learning method [4], in which inverse-docking approach plays an important role in target identification [5,6].

Inverse docking is a novel technology that can dock a compound with known biological activity into the binding sites of all 3D structures in given protein database [7]. The procedure of docking involves multiple conformer shape-matching alignment of drug molecule to a cavity followed by molecular-mechanics torsion optimization and energy minimization on both the molecule and the protein residues at the binding region [8]. And the screening is conducted by the evaluation of molecular mechanics energy, and the potential protein ‘hits’ can be selected by further analysis of binding competitiveness against other ligands that bind to the same receptor site [9]. Further, the most commonly used drug target database is potential drug target database (PDTD, http://www.dddc.ac.cn/pdtd/), which it contains 1207 entries covering 841 known and potential drug targets with structures from the Protein Data Bank (PDB) [10]. There are also a number of academic or commercial available pathway databases and network building tools, such as MetaCore™ and Integrity SM [11,12]. MetaCore™ is one of the most suitable tools for functional mining of large, inherently noisy experimental datasets, and the network visualization of drug-target, target disease and disease-gene associations can provide useful information for studies of therapeutic indications and adverse effects of drugs [13,14].

Traditional Chinese medicines (TCMs) have been used to treat many diseases for thousands of years. Dioscin, a natural steroidal saponin, exists in many Chinese medical herbs including *Dioscorea nipponica* Makino, *Dioscorea zingiberensis* C. H. Wright and *Dioscorea futschauensis* Uline. Pharmarcological studies have showed that dioscin has anti-tumor, anti-hyperlipidemic, anti-fungal and anti-virus activities [15-18]. And our previous studies showed that dioscin has significant hepatoprotective effects on carbon tetrachloride (CCl_4_) and acetaminophen induced liver damage in mice [19-21]. In the future, more and more researches of dioscin will be investigated because of its important medical value. How will the studies be defined in terms of targets priorities, biological activities, signal pathways and regulating networks affected by the compound? In routine works, the experiments with a lot of blindness should be carried out step-by-step [22,23], and they will last a long time with time consuming and laborious. Thus, a prediction of the drug targets, biological activity, signal pathways and regulatory pathways is necessary, and it will provide complementary and supporting evidence for the next experiments studies.

In the present paper, the drug-targets were predicted based on inverse docking, and enrichment analysis and network assays of dioscin were carried out by GeneGo’s MetaCore™ techniques. Some possible targets, biological activities, signal pathways and regulating networks of dioscin were predicted in advance, which should provide useful information for further investigation.

## Methods

### System

**Figure 1:**
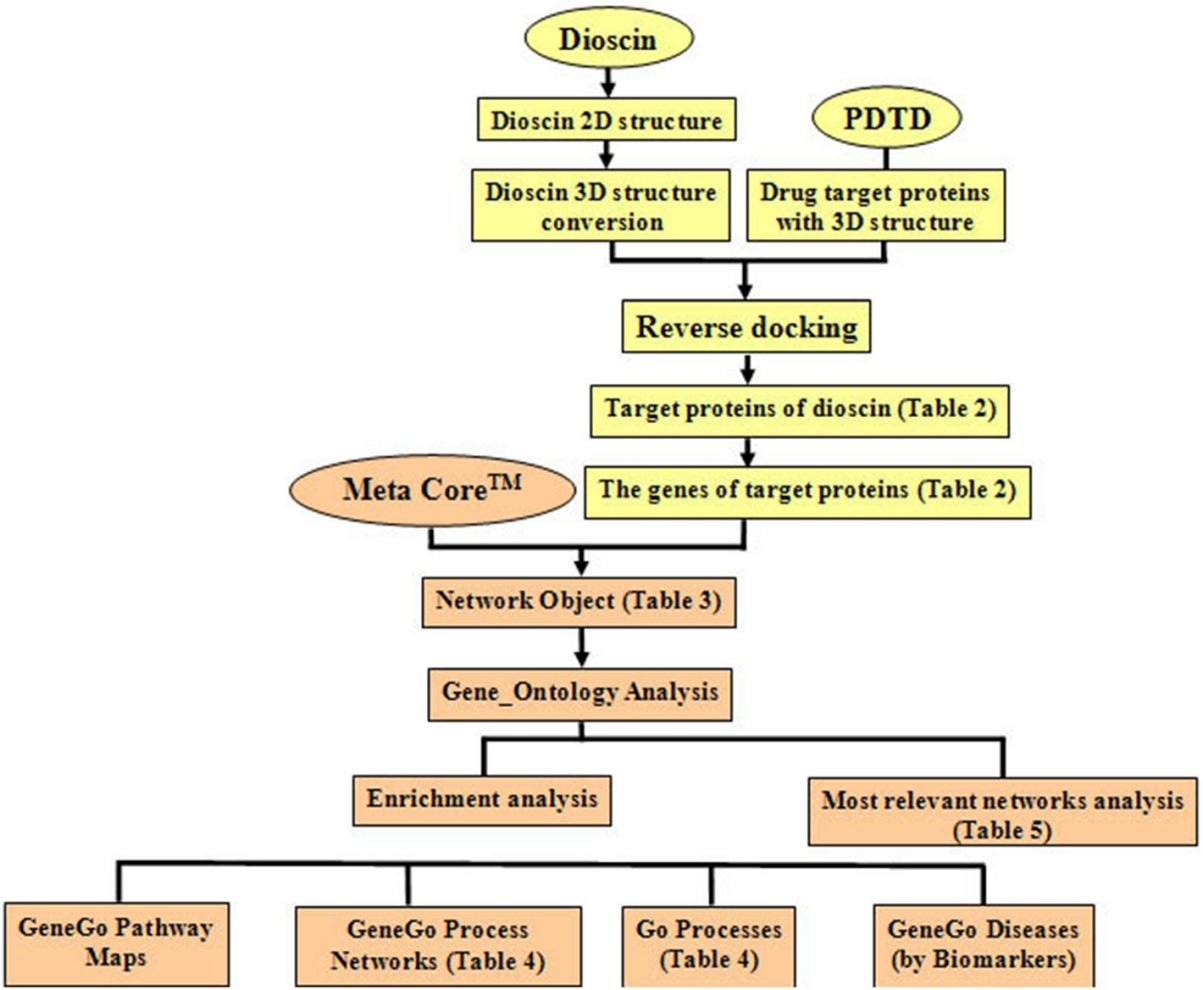
**The analysis process of the prediction of drug targets, biological activities, signal pathway and regulating networks.**

In the present paper, 2D chemical structure of dioscin was sketched using MarvinSketch (http://www.chemaxon.com), and three-dimension (3D) structure of dioscin was constructed using ISIS/Draw (ISIS/Draw, MDL Information Systems, Inc., San Leandro, CA, USA). Then, the identification and validation of all potential targets of dioscin were carried out by MDock software. The MDock is automated molecular docking software for simultaneously docking dioscin with known/available three-dimension crystal structure against drug targets from PDTD with multiple protein structure/conformations downloaded from RCSB Protein Data Bank (PDB) by using the ensemble docking algorithm [24]. After that, MetaCore platform was used to analysis the biological activities, signal pathways and regulating networks, which is a suite of software oriented toward understanding the function of gene sets discovered by expression analysis (Table 1) and based on a proprietary manually curated database of protein-protein, and protein-DNA interactions, metabolic and signaling pathways. The analysis process of the prediction of drug targets, biological activities, signal pathway and regulating networks is shown in Figure 1.

**Table 1:** **Tools and databases of MetaCore platform**

**Software/Databases**	**Website**	**Description**
MetaCore database	http://lsresearch.thomsonreuters.com/	A manually curated interactions database for >90% human protein with known function.
Gene Ontology	http://www.geneontology.org	The most often referred to publicly available protein classification based on cellular processed developed by Gene Ontology Consortium.
MetaCore, pathway module	http://lsresearch.thomsonreuters.com/maps/	A part of commercial tool MetaCore, the pathways module contains 350 interactive maps for > 2000 established pathways in human signaling, regulation and metabolism. High throughput data can be superimposed on the maps and networks built for any object.
MetaCore	http://lsresearch.thomsonreuters.com/	An integrated analytical suite based on a manually curated data of human protein-protein and protein-DNA interactions. All type of high throughput data can be used for building networks.

### Target protein screening

In the procedure of screening targets, the chemical structure of dioscin was sketched to three-dimension structure by ISIS/Draw for identifying potential biological targets. Then, the structure file was uploaded to the MDock software, the binding site analysis was applied with PDTD. In the procedure, the binding site analysis enable to identify and characterize a protein’s binding site, then use those characteristics to look for similar features in other proteins. The active site and binding energy (kcal/mol) of them were defined and calculated, when the potential drug target proteins were founded. And the threshold value of predicted binding energy was–50.0 and other options used default settings to screen the high binding target protein.

### Gene ontology analysis

#### Enrichment analysis

The corresponding gene of screened target proteins were uploaded to MetaCore platform, and the functional analyses of related data were worked out by ontology enrichment analysis based gene ontology. Here, the category of GeneGo biological processes were chosen, which includes some prebuilt molecular interaction networks, including protein-protein and protein-compounds metabolites, protein-nucleic acid interactions between all the networks.

At first, the data should be activated. When analyzing data, make sure that the threshold is set appropriately. In the analysis settings, the fold change threshold was set at 0.001, and the p-value threshold was 0.05. Namely, any genes with fold change values less than 0.001 were filtered out. Meanwhile, p-value threshold filters out genes with a p-value of more than 0.05. Other parameters also were set before analysis, such as signals was set as both, sorting method was set as statistically significant.

The enrichment analysis consists of matching gene IDs of possible targets for the “common”, “similar” and “unique” sets with gene IDs in functional ontologies in MetaCore. The probability of a random intersection between a set of IDs of the size of listed targets with ontology entities is estimated in p-value of hypergeometric intersection. The lower p-value means higher relevance of the entity to the dataset, which shows in higher rating for the entity. The ontologies include GeneGo Pathway Maps, GeneGo process Networks, Go Processes and GeneGo Diseases (by Biomarkers). The degree of relevance to different categories for the uploaded datasets is defined by p-values, and so the lower p-value gets higher priority. The distributions are calculated and showed as histograms of 10 most significant results (ranked by the-log (p-value)).

#### Most relevant networks analysis

The gene of the uploaded files is used as the input list for gene statistics analysis, the correlation between gene and network objects were obtained, and the intensity corresponds to the expression values were provided. Then, the obtained results were used for network statistics based networks built from active experiments, the relevant network objects of each network were listed, including the divergence hubs convergence hubs, edges in and edges out. The generation of biological networks uses Analyze Networks (AN) algorithm with default settings. This is a variant of the shortest paths algorithm with main parameters of relative enrichment with the uploaded data, and relative saturation of networks with canonical pathways. These networks are built on the fly and unique for the uploaded data. In this workflow the networks are prioritized based on the number of fragments of canonical pathways on the network.

## Results and discussion

### Target proteins

Inverse docking was used to identify new potential biological targets, or to identify target for components among a family of related receptors. In the present paper, 71 potential targets of dioscin identified from humans’ proteins, 7 from rats and 8 from mice were screened by MDock software. These target proteins belong to enzymes, G-protein-coupled, receptors, ion channels, and nuclear receptors, which are listed in Table 2.

**Table 2:** **The target proteins of dioscin from human, rat and mouse searched in PDTD**

**No**	**PDB**	**Uniprot_id**	**Gene symbol**	**Protein name**	**Gene id**	**Energy**
Human						
1	2old	-	-	Immune system	-	−64.3
2	1iyh	PTGD2_HUMAN	PTGDS2	Glutathione-requiring prostaglandin D synthase	27306	−65.2
3	1udu	PDE5A_HUMAN	PDE5A	cGMP-specific 3′,5′-cyclic phosphodiesterase	8654	−58
4	2ax4	PAPS2_HUMAN	PAPSS2	Bifunctional 3′-phosphoadenosine 5′-phosphosulfate synthetase 2	9060	−73.8
5	1ivo	EGFR_HUMAN	EGFR	Epidermal growth factor receptor [Precursor]	1956	−67.4
6	2a73	CO3_HUMAN	C3	Complement C3 [Precursor]	718	−67.6
7	1ivo	EGF_HUMAN	EGF	Pro-epidermal growth factor [Precursor]	1950	−67.4
8	1xbt	KITH_HUMAN	TK1	Thymidine kinase, cytosolic	7083	−71
9	1w4r	KITH_HUMAN	TK1	Thymidine kinase, cytosolic	7083	−70.2
10	1l5q	PYGL_HUMAN	PYGL	Glycogen phosphorylase, liver form	5836	−68.8
11	1pl2	GSTA1_HUMAN	GSTA1	Glutathione S-transferase A1	2938	−65.1
12	1m5o	SNRPA_HUMAN	SNRPA	U1 small nuclear ribonucleoprotein A	6626	−74
13	1tl8	TOP1_HUMAN	TOP1	DNA topoisomerase 1	7150	−55.2
14	1rr8	TOP1_HUMAN	TOP1	DNA topoisomerase 1	7150	−62.1
15	1nd6	PPAP_HUMAN	ACPP	Prostatic acid phosphatase [Precursor]	55	−57.7
16	2c5n	CCNA2_HUMAN	CCNA2	Cyclin-A2	890	−72.2
17	2c6t	CCNA2_HUMAN	CCNA2	Cyclin-A2	890	−67.4
18	2c5x	CCNA2_HUMAN	CCNA2	Cyclin-A2	890	−73.8
19	1yah	EST1_HUMAN	CES1	Liver carboxylesterase 1 [Precursor]	1066	−68.3
20	2c5n	CDK2_HUMAN	CDK2	Cell division protein kinase 2	1017	−72.2
21	2c6t	CDK2_HUMAN	CDK2	Cell division protein kinase 2	1017	−67.4
22	2c5x	CDK2_HUMAN	CDK2	Cell division protein kinase 2	1017	−73.8
23	1sk6	CYAA_BACAN	cya	Calmodulin-sensitive adenylate cyclase [Precursor]	2820138	−63.3
24	1sk6	CYAA_BACAN	cya	Calmodulin-sensitive adenylate cyclase [Precursor]	3361726	−63.3
25	2a4z	PK3CG_HUMAN	PIK3CG	Phosphatidylinositol-4,5-bisphosphate 3-kinase catalytic subunit gamma isoform	5294	−61.2
26	1sa4	FNTA_HUMAN	FNTA	Protein farnesyltransferase/geranylgeranyltransferase type-1 subunit alpha	2339	−63.5
27	1sa4	FNTB_HUMAN	FNTB	Protein farnesyltransferase subunit beta	2342	−63.5
28	1h2v	NCBP2_HUMAN	NCBP2	Nuclear cap-binding protein subunit 2	22916	−61.2
29	2uym	KIF11_HUMAN	KIF11	Kinesin-like protein KIF11	3832	−58.8
30	1ko6	NUP98_HUMAN	NUP98	Nuclear pore complex protein Nup98-Nup96 [Precursor]	4928	−74.2
31	2i6a	ADK_HUMAN	ADK	Adenosine kinase	132	−66.6
32	2fdp	BACE1_HUMAN	BACE1	Beta-secretase 1 [Precursor]	23621	−70.2
33	2ic5	RAC3_HUMAN	RAC3	Ras-related C3 botulinum toxin substrate 3 [Precursor]	5881	−75.3
34	1sk6	CALM_HUMAN	CALM1	Calmodulin	801	−63.3
35	1sk6	CALM_HUMAN	CALM1	Calmodulin	808	−63.3
36	1sk6	CALM_HUMAN	CALM1	Calmodulin	805	−63.3
37	1 m63	PPIA_HUMAN	PPIA	Peptidyl-prolyl cis-trans isomerase A	5478	−81.3
38	1 m63	PPIA_HUMAN	PPIA	Peptidyl-prolyl cis-trans isomerase A	653214	−81.3
39	1 m63	PPIA_HUMAN	PPIA	Peptidyl-prolyl cis-trans isomerase A	654188	−81.3
40	1 m63	CANB1_HUMAN	PPP3R1	Calcineurin subunit B type 1	5534	−81.3
41	1y4v	HBB_HUMAN	HBB	Hemoglobin subunit beta	3043	−79.9
42	1y4g	HBB_HUMAN	HBB	Hemoglobin subunit beta	3043	−62.1
43	1y45	HBB_HUMAN	HBB	Hemoglobin subunit beta	3043	−57.1
44	1y85	HBB_HUMAN	HBB	Hemoglobin subunit beta	3043	−55.6
45	1rq3	HBB_HUMAN	HBB	Hemoglobin subunit beta	3043	−63
46	1y46	HBB_HUMAN	HBB	Hemoglobin subunit beta	3043	−59.4
47	1y7g	HBB_HUMAN	HBB	Hemoglobin subunit beta	3043	−71.9
48	1y4q	HBB_HUMAN	HBB	Hemoglobin subunit beta	3043	−73.6
49	1y4v	HBA_HUMAN	HBA1	Hemoglobin subunit alpha	3039	−79.9
50	1y4v	HBA_HUMAN	HBA1	Hemoglobin subunit alpha	3040	−79.9
51	1y4g	HBA_HUMAN	HBA1	Hemoglobin subunit alpha	3040	−62.1
52	1y4g	HBA_HUMAN	HBA1	Hemoglobin subunit alpha	3039	−62.1
53	1y45	HBA_HUMAN	HBA1	Hemoglobin subunit alpha	3040	−57.1
54	1y45	HBA_HUMAN	HBA1	Hemoglobin subunit alpha	3039	−57.1
55	1y85	HBA_HUMAN	HBA1	Hemoglobin subunit alpha	3040	−55.6
56	1y85	HBA_HUMAN	HBA1	Hemoglobin subunit alpha	3039	−55.6
57	1rq3	HBA_HUMAN	HBA1	Hemoglobin subunit alpha	3040	−63
58	1rq3	HBA_HUMAN	HBA1	Hemoglobin subunit alpha	3039	−63
59	1y46	HBA_HUMAN	HBA1	Hemoglobin subunit alpha	3040	−59.4
60	1y46	HBA_HUMAN	HBA1	Hemoglobin subunit alpha	3039	−59.4
61	1y7g	HBA_HUMAN	HBA1	Hemoglobin subunit alpha	3040	−71.9
62	1y7g	HBA_HUMAN	HBA1	Hemoglobin subunit alpha	3039	−71.9
63	1y4q	HBA_HUMAN	HBA1	Hemoglobin subunit alpha	3040	−73.6
64	1y4q	HBA_HUMAN	HBA1	Hemoglobin subunit alpha	3039	−73.6
65	1fro	LGUL_HUMAN	GLO1	Lactoylglutathione lyase	2739	−67.4
66	1 m63	PP2BA_HUMAN	PPP3CA	Serine/threonine-protein phosphatase 2B catalytic subunit alpha isoform	5530	−81.3
67	1h2v	NCBP1_HUMAN	NCBP1	Nuclear cap-binding protein subunit 1	4686	−61.2
68	2gk6	RENT1_HUMAN	UPF1	Regulator of nonsense transcripts 1	5976	−72.9
69	2ovp	FBXW7_HUMAN	FBXW7	F-box/WD repeat-containing protein 7	55294	−63.5
70	2pfo	DPOLL_HUMAN	POLL	DNA polymerase lambda	27343	−60.7
71	2 h16	ARL5A_HUMAN	ARL5A	ADP-ribosylation factor-like protein 5A	26225	−66.6
Rat						
1	1cte	CATB_RAT	Ctsb	Cathepsin B [Precursor]	-	−65.8
2	1ewk	GRM1_RAT	Grm1	Metabotropic glutamate receptor 1 [Precursor]	24414	−68.6
3	1zzu	NOS1_RAT	Nos1	Nitric oxide synthase, brain	24598	−74.3
4	1rs6	NOS1_RAT	Nos1	Nitric oxide synthase, brain	24598	−76.2
5	1kzo	FNTB_RAT	Fntb	Protein farnesyltransferase subunit beta	64511	−67.4
6	2bed	FNTB_RAT	Fntb	Protein farnesyltransferase subunit beta	64511	−71.5
7	1kzo	FNTA_RAT	Fnta	Protein farnesyltransferase/geranylgeranyltransferase type-1 subunit alpha	25318	−67.4
Mouse						
1	2ihm	DPOLM_MOUSE	Polm	DNA polymerase mu	54125	−59.2
2	1f3a	GSTA1_MOUSE	Gsta1	Glutathione S-transferase A1	14857	−63
3	2oi9	HA1L_MOUSE	H2-L	H-2 class I histocompatibility antigen, L-D alpha chain [Precursor]	-	−62.1
4	1iea	HA21_MOUSE	H2-Ea	H-2 class II histocompatibility antigen, E-D alpha chain [Precursor]	-	−66.5
5	1fng	HA22_MOUSE	-	H-2 class II histocompatibility antigen, E-K alpha chain [Precursor]	-	−66.1
6	1qom	NOS2_MOUSE	Nos2	Nitric oxide synthase, inducible	18126	−66.2
7	2gb4	TPMT_MOUSE	Tpmt	Thiopurine S-methyltransferase	22017	−57.5
8	2oi9	TVA1_MOUSE	-	T-cell receptor alpha chain V region PHDS58 [Precursor]		

### Enrichment analysis

#### GeneGo pathway Maps

**Figure 2:**
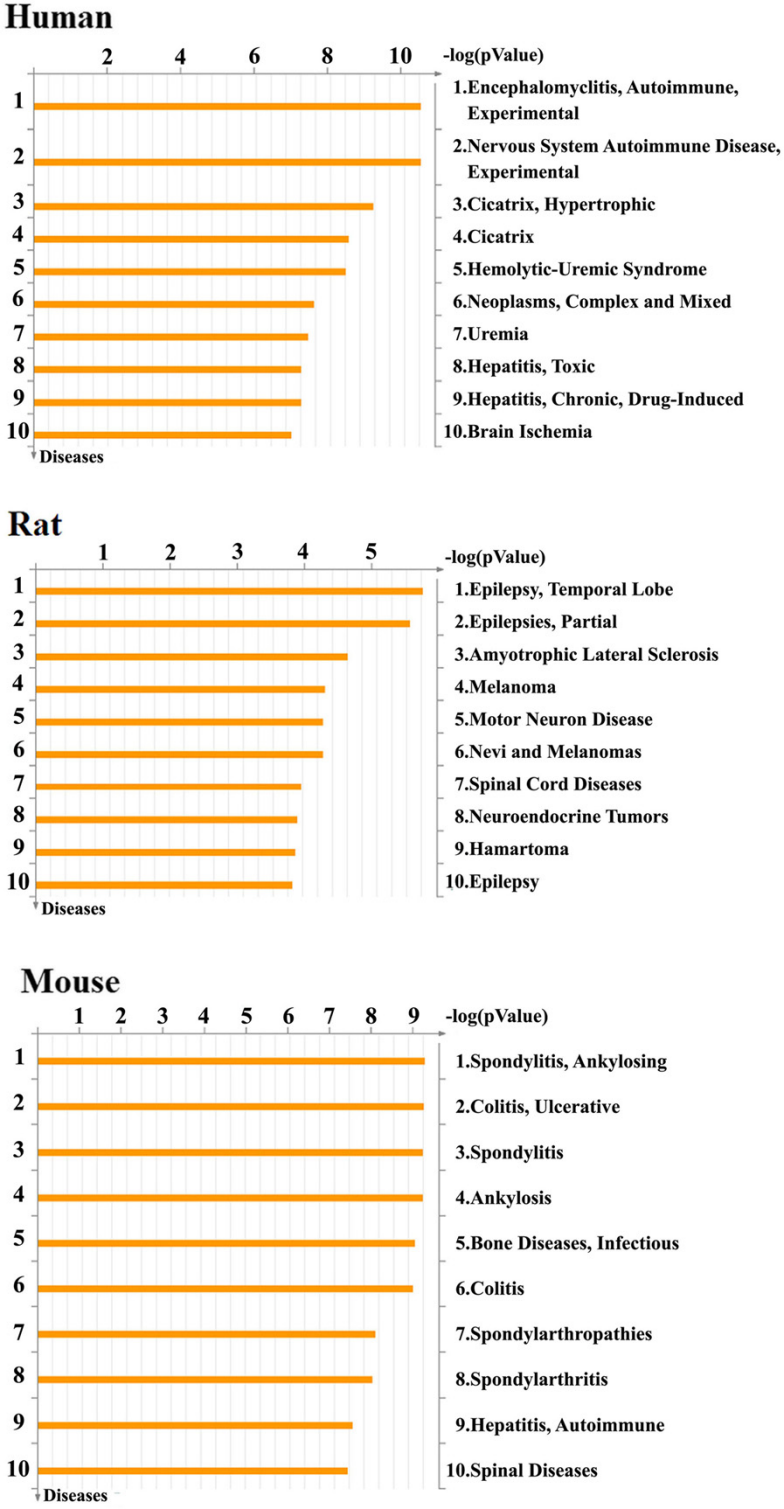
**GeneGo Diseases (by Biomarkers) of human, rat and mouse.** Sorting is done for the “Statistically significant Diseases” set.

It is generally recognized that the pathway-based analysis can provide much significant information. Canonical pathway maps represent a set of about 650 signaling and metabolic maps covering human biology (signaling and metabolism) in a comprehensive way. The profile of network objects (Table 3) was uploaded to search canonical pathway maps. All maps are drawn from scratch by GeneGo annotators and manually curated & edited. From the distributions shown in Figure 2, the most significantly multistep pathways from literature consensus were enriched in the data set. Experimental data is visualized on the maps as yellow (for down-regulation) histograms. The height of the histogram corresponds to the relative expression value for a particular gene/protein.

**Table 3:** **The Network objects and its functions**

**No**	**Species**	**Gene Name**	**Network Objects**	**Protein Function**
1	Homo sapiens	ACPP	PPAP	Protein phosphatase
2	Homo sapiens	ADK	Adenosine kinase	Generic kinase
3	Homo sapiens	ARL5A	ARL5	RAS superfamily
4	Homo sapiens	BACE1	BACE1	Generic protease
5	Homo sapiens	C3	C3	Generic binding protein
			C3a	Generic binding protein
			C3b	Generic binding protein
			C3c	Generic binding protein
			C3dg	Generic binding protein
			C5 convertase (C2aC4bC3b)	Generic protease
			C5 convertase (C3bBb)	Generic protease
			iC3b	Generic binding protein
6	Homo sapiens	CALM1	Calmodulin	Generic binding protein
7	Homo sapiens	CALM2	Calmodulin	Generic binding protein
8	Homo sapiens	CALM3	Calmodulin	Generic binding protein
9	Homo sapiens	CCNA2	Cyclin A	Generic binding protein
			Cyclin A2	Generic binding protein
10	Homo sapiens	CDK2	CDK2	Protein kinase
11	Homo sapiens	CES1	CES1	Generic enzyme
12	Homo sapiens	EGF	EGF	Receptor ligand
13	Homo sapiens	EGFR	EGFR	Receptor with enzyme activity
14	Homo sapiens	FBXW7	Cul1/Rbx1 E3 ligase	Generic enzyme
			FBXW7	Generic binding protein
			Skp2/TrCP/FBXW	Generic binding protein
15	Homo sapiens	FNTA	FTase	Generic enzyme
			FTase-alpha	Generic enzyme
			GGTase-I	Generic enzyme
16	Homo sapiens	FNTB	FTase	Generic enzyme
			FTase-beta	Generic enzyme
17	Homo sapiens	GLO1	Glyoxalase I	Generic enzyme
18	Homo sapiens	GSTA1	GSTA1	Generic enzyme
19	Homo sapiens	HBA1	Adult hemoglobin	Generic protein
			Alpha1-globin	Transporter
			HP/HB complex	Generic protein
20	Homo sapiens	HBA2	Adult hemoglobin	Generic protein
			Alpha1-globin	Transporter
			HP/HB complex	Generic protein
21	Homo sapiens	HBB	Adult hemoglobin	Generic protein
			HBB	Transporter
			HP/HB complex	Generic protein
22	Homo sapiens	HPGDS	PGDS	Generic enzyme
23	Homo sapiens	KIF11	KNSL1	Generic binding protein
24	Homo sapiens	NCBP1	CBP80	Generic binding protein
25	Homo sapiens	NCBP2	CBP20	Generic binding protein
26	Homo sapiens	NUP98	NUP98	Generic channel
			NUP98/HHEX fusion protein	Transcription factor
			NUP98/HOXA9 fusion protein	Transcription factor
			Nuclear pore complex proteins	Generic channel
27	Homo sapiens	PAPSS2	PAPSS2	Generic kinase
28	Homo sapiens	PDE5A	PDE	Generic protein
			PDE5A	Generic enzyme
29	Homo sapiens	PIK3CG	PI3K cat class IB (p110-gamma)	Lipid kinase
30	Homo sapiens	POLL	DNA polymerase lambda	Generic enzyme
31	Homo sapiens	PPIA	Cyclophilin A	Generic enzyme
32	Homo sapiens	PPP3CA	Calcineurin A (alpha)	Protein phosphatase
			Calcineurin A (catalytic)	Protein phosphatase
33	Homo sapiens	PPP3R1	Calcineurin B (regulatory)	Generic binding protein
			Calcineurin B1	Generic binding protein
34	Homo sapiens	PYGL	Glycogen phosphorylase	Generic enzyme
			PYGL	Generic enzyme
35	Homo sapiens	RAC3	Rac3	RAS superfamily
36	Homo sapiens	SNRPA	SNRPA	Generic binding protein
37	Homo sapiens	TK1	TK1	Generic kinase
38	Homo sapiens	TOP1	TOP1	Generic enzyme
39	Homo sapiens	UPF1	RENT1	Generic binding protein
1	Rattus norvegicus	Ctsb	Cathepsin B	Generic protease
2	Rattus norvegicus	Fnta	FTase	Generic enzyme
			FTase-alpha	Generic enzyme
			GGTase-I	Generic enzyme
3	Rattus norvegicus	Fntb	FTase	Generic enzyme
			FTase-beta	Generic enzyme
4	Rattus norvegicus	Grm1	Galpha(q)-specific metabotropic glutamate GPCRs	Generic receptor
			mGluR1	GPCR
5	Rattus norvegicus	Nos1	nNOS	Generic enzyme
1	Mus musculus	Gsta1	Gsta1 (mouse)	Generic enzyme
2	Mus musculus	H2-D1	HLA-B	Generic receptor
			MHC class I	Generic receptor
3	Mus musculus	H2-Ea	HLA-DRA1	Generic receptor
			MHC class II	Generic receptor
			MHC class II alpha chain	Generic receptor
4	Mus musculus	H2-Ea	HLA-DRA1	Generic receptor
			MHC class II	Generic receptor
			MHC class II alpha chain	Generic receptor
5	Mus musculus	H2-L	H-2 L(d)	Generic receptor
			HLA-C	Generic receptor
			MHC class I	Generic receptor
6	Mus musculus	Nos2	iNOS	Generic enzyme
7	Mus musculus	OTTMUSG00000015050	Trav9d-4	Generic receptor
8	Mus musculus	Polm	DNA polymerase mu	Generic enzyme
9	Mus musculus	Tpmt	Thiopurine S-methyltransferase	Generic enzyme
10	Mus musculus	Uap1	UAP1	Generic enzyme

**Figure 3:**
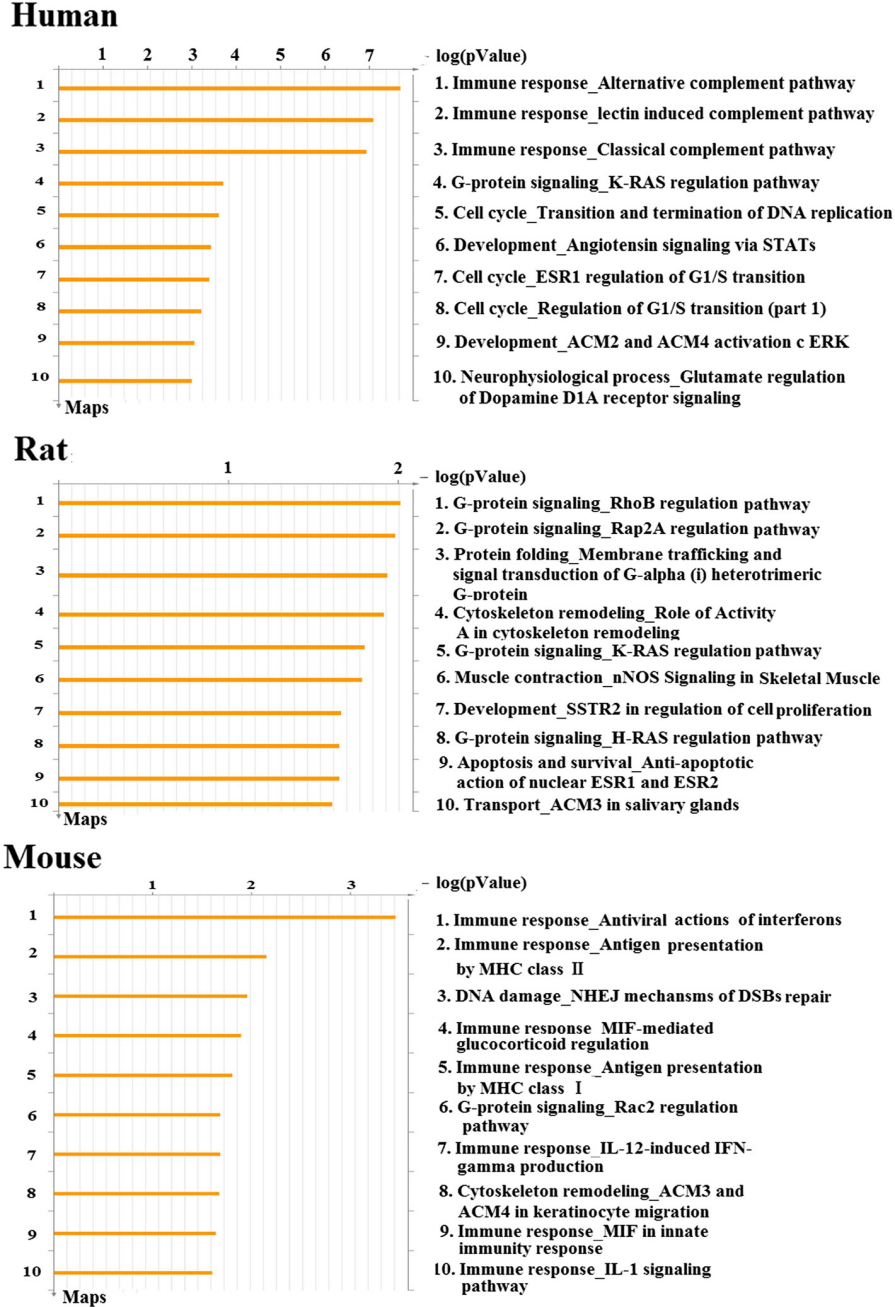
**GeneGo Pathway Maps.** Sorting is done for the “Statistically significant Maps” set.

Top scored pathway maps were sorted by statistically significant Maps (Figure 3). The top scored pathway maps were immune response alternative complement pathway, G-protein signaling_RhoB regulation pathway and immune response antiviral actions of interferons, respectively. Experimental data from all files is linked to and visualized on the maps as thermometer-like figures. Up-ward thermometers have red color and indicate up-regulated signals and down-ward (blue) ones indicate down-regulated expression levels of the genes.

Complement system can protect the host from microorganisms [25], and the alternative pathway can be directly activated by invading microorganisms. C3/C5 convertases which are complex enzymes transiently assembled on the surface of biological organisms upon activation of the complement system [26]. The generation of active C3/C5 convertases help opsonize, kill, and clear bacteria, parasites and pathogens by eliciting cellular functions including phagocytosis and inflammation [27]. In the map of human, it is initiated by the spontaneous hydrolysis of C3 which is a major effector of humoral branch of the complement system. The down-regulation of C3 treated by dioscin contributes to the down-regulation of C3a and C3b, and then they induce the down-regulation of C5 convertase. For the linkage effect, the cleavage fragments binding to specific receptors suffer affection, including CR1, C3aR, alpha-M/beta-2 integrin, alpha-X/beta-2 intetrin and CD21.

In the map of RhoB regulation pathway, RhoB is a member of small GTPases family and can control multiple cellular processes, including actin and microtubule dynamics, gene expression, cell cycle, cell polarity and membrane transport. Their abilities are bound to numerous downstream effectors which lead to diverse parallel downstream signaling pathways [28]. There are several classes of regulatory proteins affect the activation of RhoB. Among them, GGTase-I (Geranylgeranyltrans-ferase type I) and FTase (Farnesyltransferase CAAX box) promote post-translational modification of RhoB (Ras homolog gene family, member A) protein by geranylgeranylation and farnesylation, which are essential for the biological activity of RhoB. In the prediction, down-regulated expression of RhoB gene was induced by down-regulations of GGTase-I and FTase treated with dioscin.

In the map of immune response antiviral actions of interferons, iNOS (inducible NO synthase) was the network object. iNOS generates copious amounts of NO presumably to help kill or inhibit the growth of invading microorganisms or neoplastic tissue [29]. Over-expression of iNOS, a common phenomenon during chronic inflammatory conditions, generates sustainable amounts of NO. Its reactive intermediates are mutagenic, causing DNA damage or impairment of DNA repair. Recent studies also implicated NO as a key signaling molecule which can regulate the processes of tumorigenesis. Increased expression of iNOS is involved in tumors of the colon, lung, oropharynx, reproductive organs, breast, and CNS (Central Nervous System) [30]. Thus, the map indicated that dioscin can down regulate the expression level of iNOS gene. Namely, it may be a selective inhibitor of iNOS for chemoprevention of cancer.

#### GeneGo process networks and Go processes

In the GeneGo process networks analysis, sorting is done for the ‘Statistically significant Networks’ set. There are about 110 cellular and molecular processes whose content is defined and annotated by GeneGo. According to the experimental data (Table 3), ten processes networks with lower p-value were obtain (Table 4). In Go processes, the original Gene Ontology (GO) cellular processes, represented at GeneGo were included. Since most of GO processes have no gene/protein content, the “empty terms” are excluded from p-value calculations, and ten processes with lower p-values were obtain (Table 4). The results are all consistent with GeneGo pathway maps, they were associated with immune response, inflammation and cell cycle signaling, the Go processes include regulation of immune response, DNA replication, RNA transport, protein amino acid famesylation, and regulation of cell killing etc. . GeneGo Diseases (by Biomarkers).

**Table 4:** **Ten GeneGo process networks and Go processes with lower p-values**

**GeneGo process network**			
	**Network Objects**	**Name**	**p-value**
Human	51	Development_Hemopoiesis, Erythropoietin pathway	0.000003178
		Inflammation_Complement system	0.00002487
		Cell cycle_G2-M	0.00004606
		Signal transduction_WNT signaling	0.0001901
		Signal Transduction_TGF-beta, GDF and Activin signaling	0.0007943
		Cell cycle_G1-S	0.001089
		Cell cycle_Meiosis	0.00169
		Cell cycle_G1-S Growth factor regulation	0.00215
		Muscle contraction_Nitric oxide signaling in the cardiovascular system	0.00228
		Protein folding_Folding in normal condition	0.002583
Rat	7	Neurophysiological process_Transmission of nerve impulse	0.0005214
		Signal Transduction_TGF-beta, GDF and Activin signaling	0.006777
		Reproduction_GnRH signaling pathway	0.008233
		Development_Neurogenesis: Synaptogenesis	0.009411
		Cell adhesion_Synaptic contact	0.009819
		Reproduction_Gonadotropins regulation	0.01165
		Neurophysiological process_Taste signaling	0.02996
		Cell cycle_G0-G1	0.05999
		Neurophysiological process_Circadian rhythm	0.0641
		Neurophysiological process_Long-term potentiation	0.06901
Mouse	11	Immune_Antigen presentation	0.0002078
		Immune_Phagosome in antigen presentation	0.0004253
		Immune_Innate immune response to RNA viral infection	0.001665
		Inflammation_NK cell cytotoxicity	0.00532
		Muscle contraction_Relaxin signaling	0.05991
		Transport_Iron transport	0.06608
		Immune_Th17-derived cytokines	0.06881
		Inflammation_IFN-gamma signaling	0.07697
		Inflammation_Interferon signaling	0.07697
		Muscle contraction_Nitric oxide signaling in the cardiovascular system	0.08035
Go processes			
	Network Objects	Name	pValue
Human	51	DNA replication	9.331E-09
		protein localization in nucleus	6.994E-08
		mRNA transport	1.706E-07
		protein amino acid farnesylation	1.928E-07
		positive regulation of biological process	2.181E-07
		establishment of RNA localization	2.994E-07
		nucleic acid transport	2.994E-07
		RNA transport	2.994E-07
		RNA localization	0.000000357
		protein farnesylation	3.848E-07
Rat	7	protein amino acid farnesylation	4.46E-10
		protein farnesylation	8.918E-10
		positive regulation of nitric-oxide synthase 2 biosynthetic process	1.56E-09
		regulation of nitric-oxide synthase 2 biosynthetic process	2.496E-09
		protein amino acid prenylation	9.799E-09
		protein prenylation	1.274E-08
		regulation of sensory perception of pain	1.455E-07
		regulation of sensory perception	1.455E-07
		synaptic transmission	3.092E-07
		transmission of nerve impulse	6.724E-07
Mouse	11	antigen processing and presentation of exogenous peptide antigen	4.316E-11
		antigen processing and presentation of exogenous antigen	9.457E-11
		antigen processing and presentation of peptide antigen	2.176E-10
		antigen processing and presentation	4.237E-09
		regulation of immune response	1.693E-08
		positive regulation of leukocyte mediated cytotoxicity	1.706E-08
		positive regulation of cell killing	2.148E-08
		positive regulation of immune system process	2.238E-08
		regulation of leukocyte mediated cytotoxicity	4.251E-08
		regulation of cell killing	5.109E-08

MetaCore can be used for uploading experimental data (Table 3) to discover and validate biomarker. Using bioinformatics approaches, numerous candidate biomarkers associated with the development or prognosis of human disease were reported. Disease folders represent over 500 human diseases with gene content annotated by GeneGo. Disease folders are organized into a hierarchical tree.

**Figure 4:**
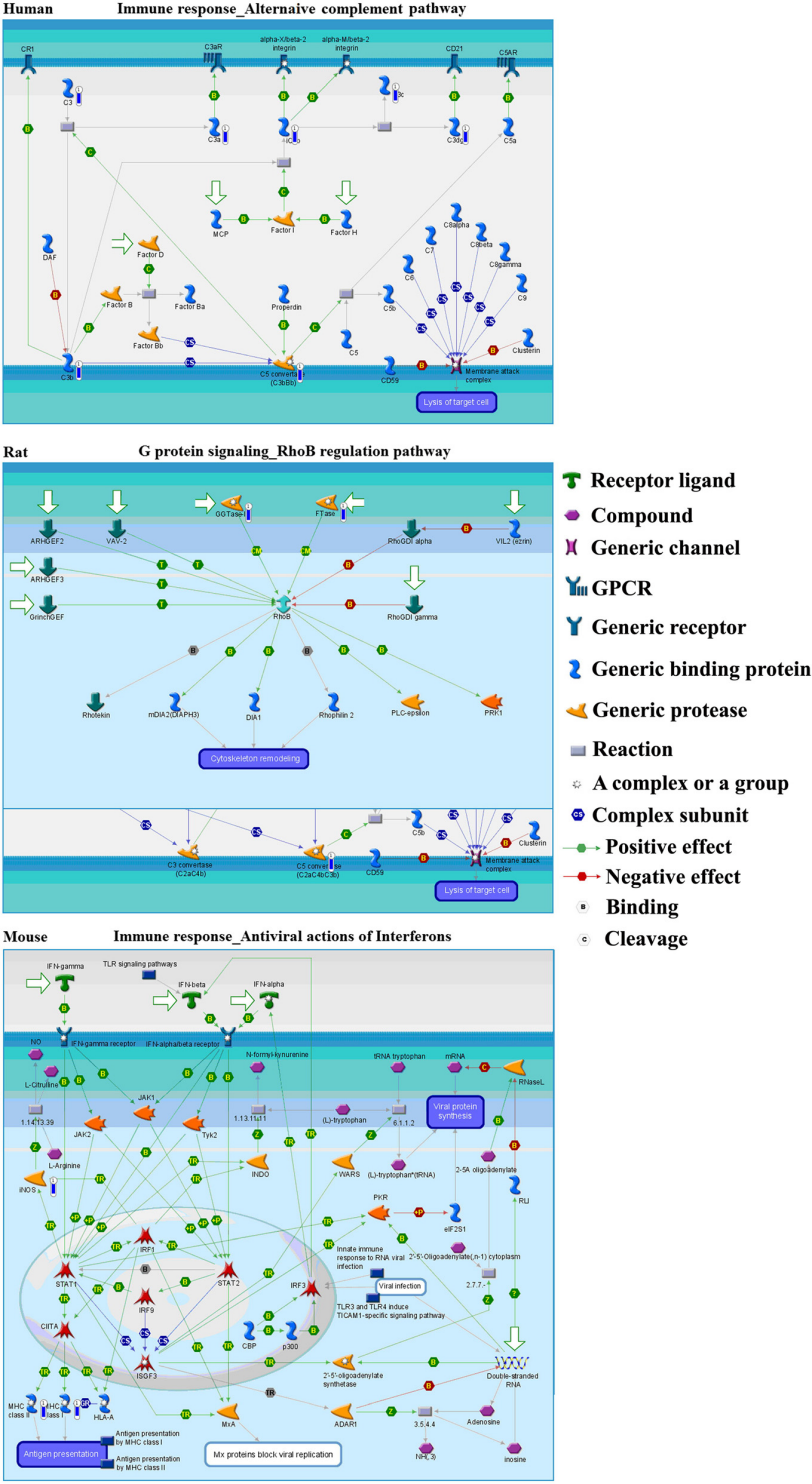
**The top scored map (map with the lowest p-value) of human, rat and mouse based on the enrichment distribution sorted by ‘Statistically significant Maps’ set.** Experimental data from all files is linked to and visualized on the maos as thermometer-like figures. Up-ward thermometers have red color and indicate up-regulated signals and down-ward (blue) ones indicate down regulated expression levels of genes.

In the paper, the enriched disease was detected by the biomarkers. Using network objects known (Table 3) to be associated with dioscin as set of interest, the frequency was recomputed by summing object occurrences for disease. Then, p-values were obtained, which assumes that the probability of picking a network objects annotated with a disease in the reference set. The results are shown in Figure 4. Gene contents may be different greatly between two complex diseases such as cancers and Mendelian diseases. Also, coverages of different diseases in literature are skewed. The two factors may affect p-value prioritization.

For human, dioscin may associate itself with experimental autoimmune encephalomyelitis, experimental nervous system autoimmune disease, hypertrophic cicatrix, cicatrix, hemolytic-uremic syndrome, complex and mixed neoplasms, uremia, toxic hepatitis, drug-induced chronic hepatitis, brain ischemia. For rat, dioscin may associate itself with temporal lobe epilepsy, partial epilepsies, amyotrophic lateral sclerosis, melanoma, motor neuron disease, nevi and melanomas, spinal cord diseases, neuroendocrine tumors, hamartoma and epilepsy. For mouse, dioscin may associate itself with ankylosing spondylitis, ulcerative colitis, spondylitis, ankylosis, infectious bone diseases, colitis, spondylarthropathies, spondylarthritis, autoimmune hepatitis, spinal diseases.

### The most relevant networks

The network analysis can provide primary information about physical connectivity and functional relationships between proteins/genes. MetaCore database is suitability for manually curated interactions database over 90% human proteins with known function [31]. MetaCore has four “Analyze” network algorithms which are useful when we have a large number of network objects. Among them, analyze network creates a large network and breaks it up into smaller sub-networks which are all ranked by p-value. And analyze transcription regulation works in a similar way. The other two “Analyze” network algorithms (transcription factors and receptors) focus on the presence of either start-nodes or end-nodes of a certain pathway. In the paper, the biological networks were created by Analyze networks algorithm, and the related objects used for network building are listed in Table 3.

As all objects on the networks are annotated, they can be associated with one or more cellular functions including DNA repair, cell cycle or apoptosis. The networks can be scored and prioritized based on statistical “relevance” in the function processes and maps. Each network is associated with a g-score and p-value. The priority can be defined as a proportion of the nodes with the data to the total number of nodes on the networks measured with z-score value. In general, a high positive g-score means it is highly saturated with genes from the experiment data.

**Figure 5:**
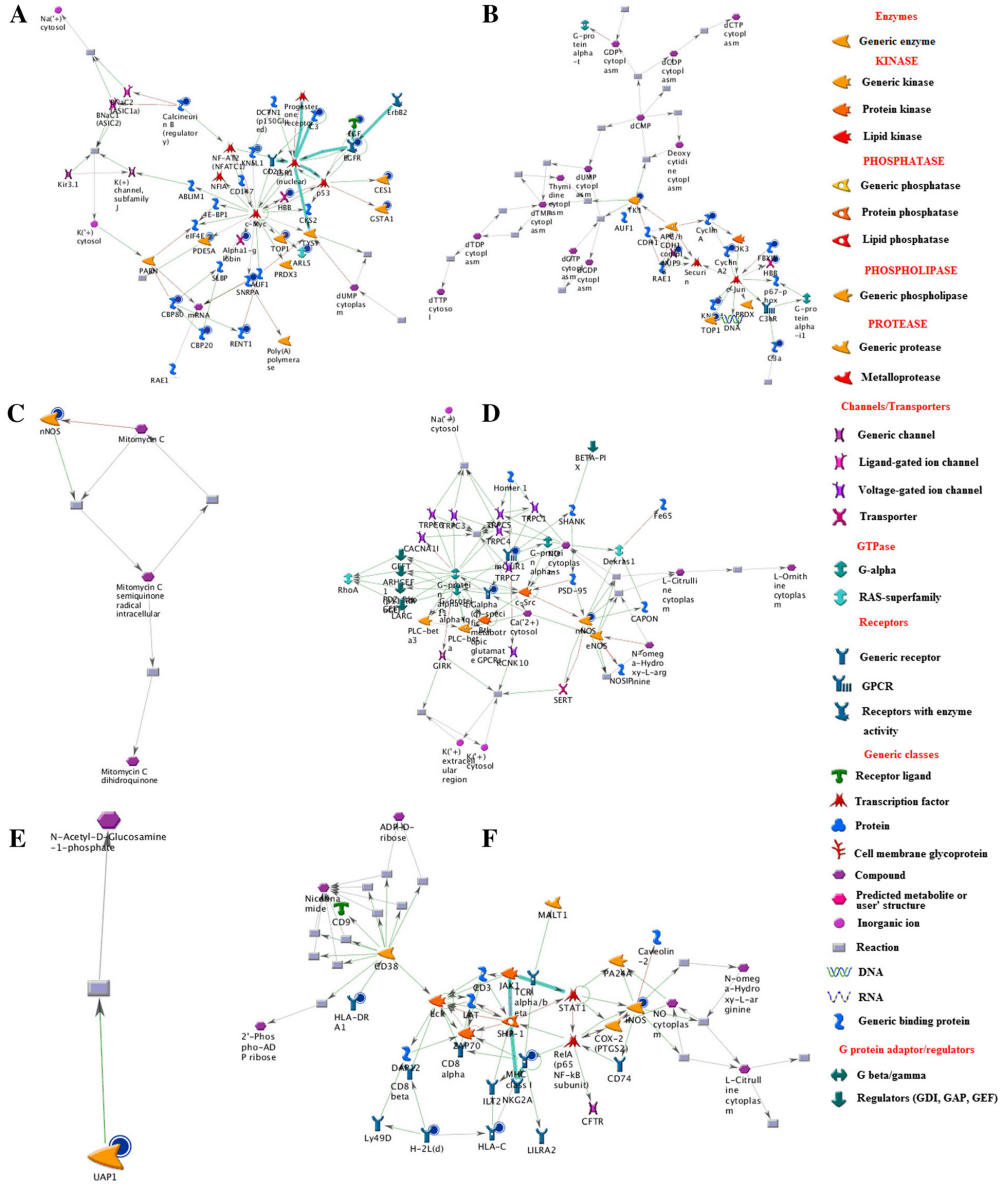
**The top two networks scored by MetaCore (AN network) of human (A and B), rat (C and D) and mouse (E and F). A:** mRNA metabolic process, RNA metabolic process and response to chemical stimulus of human; **B:** cell division, mitosis and nuclear division; **C:** peptidyl-cysteine S-nitrosylation, drug catabolic process and exogenous drug catabolic process; **D:** regulation of metal ion transport, ion transport and negative regulation of potassium ion transport; **E:** N-acetylglucosamine biosynthetic, UDP-N-acetylglucosamine biosynthetic and glucosamine biosynthetic process; **F:** positive regulation of immune system, regulation of immune system and response to stimulus. Thick cyan lines indicate the fragments of canonical pathways. Upregulated genes are marked with red circles; downregulated with blue circles. The ‘checkerboard’ color indicates mixed expression for the gene between files or between multiple tags for the same gene.

The g-score, p-values and z-score of networks are listed in Table 5, and the top two networks of each species are shown in Figure 5. Relative intensity corresponds to the expression value. Unregulated genes are marked with red circles, while down regulated genes with blue circles. The ‘checkerboard’ color indicates mixed expression for the gene between files or between multiple tags for the same gene.

**Table 5:** **g-score, z-score and p-value of the most relevant networks**

**No**	**Process**	**Size**	**Target**	**p-value**	**z-score**	**g-score**
Human						
1	mRNA metabolic process (19.5%),	50	16	4.35e-39	80.59	93.09
	RNA metabolic process (29.3%),					
	response to chemical stimulus (41.5%)					
2	cell division (40.0%),	50	9	1.72e-21	55.79	55.79
	mitosis (35.0%),					
	nuclear division(35.0%)					
3	glycogen metabolic process (23.1%),	50	9	2.39e-21	54.90	54.90
	cellular glucan metabolic process (23.1%),					
	glucan metabolic process (23.1%)					
4	intracellular signaling cascade (51.3%),	50	11	2.32e-22	50.84	52.09
	signal transduction (71.8%),					
	biological regulation (97.4%)					
5	DNA metabolic process (40.5%),	50	9	1.15e-19	45.25	51.50
	cellular response to DNA damage stimulus (31.0%),					
	response to DNA damage stimulus (31.0%)					
Rat						
1	peptidyl-cysteine S-nitrosylation (100.0%),	7	1	2.54e-04	62.68	62.68
	drug catabolic process (100.0%),					
	exogenous drug catabolic process (100.0%)					
2	regulation of metal ion transport (26.5%),	50	3	1.78e-08	39.59	39.59
	regulation of ion transport (26.5%),					
	negative regulation of potassium ion transport (14.7%)					
3	No processes found	4	0	1.00e + 00	0.00	0.00
Mouse						
1	N-acetylglucosamine biosynthetic process (100.0%),	3	1	1.82e-04	74.17	74.17
	UDP-N-acetylglucosamine biosynthetic process (100.0%),					
	glucosamine biosynthetic process (100.0%)					
2	positive regulation of immune system process (44.8%),	50	5	1.42e-14	64.51	65.76
	regulation of immune system process (51.7%),					
	regulation of response to stimulus (51.7%)					
3	positive regulation of Schwann cell differentiation (50.0%),	16	1	5.45e-04	42.81	42.81
	regulation of Schwann cell differentiation (50.0%),					
	response to cobalamin (50.0%)					
4	response to other organism (100.0%),	5	1	9.08e-04	33.15	33.15
	response to biotic stimulus (100.0%),					
5	transepithelial chloride transport (33.3%),	9	1	1.09e-03	30.26	30.26
	transepithelial transport (33.3%),					
	somatic hypermutation of immunoglobulin genes (33.3%)

The network (p=4.35e^-39^, g-score=93.09) resulting from the experiment data is shown in Figure 5A. The c-Myc is divergence hub, and the ESR1 (nuclear) is a convergence hub in the network. The c-Myc protein is a key transcriptional factor, and it is almost universally involved in cell cycle progression, transformation and apoptosis through targeting of downstream genes [32]. ESR1 (Arabidopsis Enhancer of Shoot Regeneration 1) was identified as a gene that enhance the in vitro shoot regeneration efficiency when over-expressed [33]. The network includes 16 possible targets including ARL5, TOP1, HBB, KNSL1, CBP80, CBP20, Calcineurin B, RENT1, SNRPA, CSTA1, CES1, C3, EGFR, EGF, Alphal-globin and PDESA. EGF is a metastatic inducer of tumor cells, which activates epidermal growth factor receptor (EGFR)-induced signal pathway to induce cancer metastasis [34]. CES1 is the most versatile human carboxylesterase, and it plays critical roles in drug metabolism and lipid mobilization. Excessive induction of CES1 provides a mechanism for potential anti-oxidants protective effect on human health [35]. The cap-binding protein heterodimer CBP80-CBP20 initially undergo a pioneer round of translation of newly synthesixed messenger ribonucleoproteins (mRNPs) [36]. C3 is a main complement in complement pathway [37]. TOP1 unwinds DNA by making transient single strand breaks that relieves the tosion of supercoiled DNA.

The network (p=41.72e^−21^, g-score=55.79) is shown in Figure 5B, which contains 9 of drug targets, including Cyclin A, Cyclin A2, HBB, C3a, TOP1, KNSL1, NUP98, TK1 and FBSW7. The divergence hub of the network was c-Jun, and the convergence hubs were c-Jun, APC/Hcdh1 complex and dTMP cytoplasm. The c-Jun N-terminal kinase (JNK) signaling pathway plays a critical role in inflammation _ complement system [38]. JNK can be activated by exposure of cells to cytokines or environmental stress, indicating that this signaling pathway may contribute to inflammatory responses [39]. And genetic and biochemical studies demonstrate that this signaling pathway also regulates cellular proliferation, apoptosis and tissue morphogenesis [40].

The networks of rat are shown in Figure 5C and D, and the networks of mouse are shown in Figure 5E and F. From the networks, well connected clusters of root nodes were found, and more flexibility in the connection were presented. In Figure 5C, mitomycin C can treat a variety of malignancies, such as head and neck cancers and superficial transitional cell carcinoma of the bladder [41]. The nNOS and mitomycin C is involved in a signal pathway, nNOS is down regulated by dioscin while mitomycin C is up-regulation. Thus, dioscin could be used to anti-cancer through that pathway. In Figure 5E, N-acetylglucosamine-1-phosphate catalyzes the formation of UDP-GlcNAC, which is an essential precursor of petidoglycan and the rhamnose-GlcNAc linker region of mycobacterial cell wall [42]. Thus, dioscin may be a potential anti-infections drug through down-regulation of UAP1.

All those results indicated that dioscin may exert biological effects through multi-channel. Such as, dioscin is a TOP1 inhibitor, inhibits relegation and stabilizes the DNA-TOP1 complex in the cleaved DNA form, ultimately leading to breaks of DNA chains and cell death. Thus, the dioscin could used to treat cancer though the cell cycle–transition and termination of DNA replication pathway. And it could inhibit cancer metastasis through EGFR-induced signal pathway. In addition, it could be used to treat inflammation though JNK signaling pathway. However, the dioscin may induce some side effect by down-regulation of complement system.

## Conclusions

In the paper, we presented an application of in-*silico* inverse docking technique coupled with bioinformatics approach to predict the possible targets, biological activities, signal pathways and regulating networks of dioscin. Those studies provide valuable information for future in vitro and in vivo works to validate the previous in silico findings.

## Availability and supporting data

MetaCore is available at http://www.genego.com.
